# Clinicopathological Study of Mucormycosis at Varied Sites During the COVID-19 Pandemic

**DOI:** 10.7759/cureus.71815

**Published:** 2024-10-18

**Authors:** Ramya Potti, Anusha Mullagura, Inuganti Venkata Renuka, Rizwana Shaik, A. Alluri Bhavani

**Affiliations:** 1 Pathology, NRI Medical College, Guntur, IND

**Keywords:** covid, diabetes, histopathology, mucormycosis, zygomatic

## Abstract

Background: Mucormycosis, also known as black fungus, is a rare but serious fungal infection caused by mucor that belongs to Zygomycotic species. Mucor is characterized by non-septate, irregularly wide hyphae with right-angle branching. Mucor can infect different systems of the body and manifest differently depending on the location of the infection, which includes pulmonary, gastrointestinal, rhino-cerebral, and cutaneous.

Aim: The study aims to analyze the incidence, clinical history, and microscopic features associated with mucormycosis in the COVID-19 pandemic at our institute.

Materials and methods: This is a prospective study conducted in the Department of Pathology for three years from March 2020 to April 2022. The samples were collected from different clinical departments mostly from Surgery and Radiology. These samples were fixed in 10% formalin, processed, stained with hematoxylin and eosin (H&E) and were analysed.

Results: A total of 69 cases were included in the study. The age range was 25-80 years and male preponderance was seen with an M:F ratio of 2.6:1. Diabetes was the most common risk factor seen in 53 (77%) cases followed by post-COVID-19 status in 49 (71%) cases. The most common site was paranasal sinuses (58, 84%) followed by orbit (eight, 11%) and two (2.8%) cases each of lung and bone. Fifty (72.4%) cases showed necrosis and 10 (14.5%) cases showed granulomatous inflammation. Angioinvasion was seen in 27 (39%) cases, bony invasion was seen in 26 (37.6%) cases and perineural invasion was seen in one (1.4%) case. We had three (4.3%) cases of mixed fungal infection (mucor and Aspergillus).

Conclusion: This study describes the clinical site of presentation and histopathological features of mucormycosis.

## Introduction

Mucormycosis, also known as black fungus, is a rare but serious fungal infection caused by mucor that belongs to Zygomycotic species. It has a subtle onset of disease [1]. Mucormycosis is one of the common fungal infections following those caused by Aspergillus and Candida [2]. These molds are commonly found in the environment, particularly in soil and decaying organic matter. Individuals with weakened immune systems or underlying health conditions such as uncontrolled diabetes, those taking certain medications, and organ transplantation are at higher risk of developing mucormycosis [3]. Mucormycosis typically disseminates via inhalation of spores or through contact with compromised skin [4]. The extent of infection transmission largely hinges on the thermal resilience of these spores. Acting as the primary line of defense, macrophages initiate phagocytosis, engulfing and neutralizing spores through oxidative mechanisms [5]. Mucor exhibits non-septate, irregularly broad hyphae featuring distinctive wide-angle branching patterns. These hyphae are identifiable in paraffin sections by using hematoxylin and eosin (H&E) stain or by specialized staining techniques such as periodic acid-Schiff (PAS) and Giemsa stains [6].

Mucor has the potential to invade various body systems including pulmonary, gastrointestinal, rhino-cerebral, and cutaneous [5,7,8]. In individuals with severe immunosuppression, such as those with compromised immune function, the fungus primarily targets the lungs [6]. Among diabetics with poorly controlled blood sugar levels, the fungus commonly spreads to the orbit and brain via nasal sinuses leading to the onset of rhinocerebral mucormycosis [6]. Regardless of its pathway, mucor induces necrosis of tissue, infiltrates blood vessels resulting in thrombosis and tissue infarction [5], and can lead to dissemination of infection throughout the body. Recently, the incidence of mucormycosis cases has surged due to risk factors, such as diabetes, post-COVID-19 conditions, and immunosuppression. The study aims to analyze the incidence, clinical history, and microscopic features associated with mucormycosis in the COVID-19 pandemic at our institute.

## Materials and methods

Our study was a prospective study conducted in the Department of Pathology for a period of two years from March 2020 to February 2022. The samples were collected from different clinical departments like Surgery and Radiology. All the relevant clinical history which includes diabetes, COVID-19 status, drug history, duration, signs and symptoms, site of involvement and radiological imaging data were analysed. These samples were fixed in 10% formalin and processed and H&E staining was done. Other tests like potassium hydroxide (KOH) mount test, special stains like Gomori methenamine silver stain (GMS), and culture were done if necessary. The sample preparations were studied for microscopic details like composition of inflammatory infiltrate, response, degree of tissue necrosis, and angioinvasion. The sample size in our study was 69 cases (the study done by Bala et al. [9], in which the sample size was 39). The statistical analysis was calculated by percentage and mean. Inclusion criteria were all cases that were biopsy-proven. Exclusion criteria were cases that were negative on histopathological examination, special stain and culture were excluded.

## Results

A total of 69 cases were included in this study. The age range was 25-80 years, the youngest was 28 years and the oldest was 80 years. In the 69 cases, 50 were males and 19 were females. There was a male preponderance and the M:F ratio was 2.6:1. 

Out of 69 cases examined, diabetes mellitus emerged as the most prevalent comorbidity present in 53 (77%) cases following closely behind were post-COVID-19 cases totaling 49 (71%), while COVID-19 status itself was identified in six (9%) cases.

Mucormycosis was seen in different sites in this study. The common site was paranasal sinuses which constituted 58 (84%) cases in which maxillary sinus was the commonest followed by ethmoid and sphenoid sinuses. The other sites were orbit (8, 11%), lung and bone each constituting two (2.8%) cases, one (1.4%) case each of the brain, small intestine, post cricoid, nasopharynx, scapula, pterygopalatine fossa, hard palate. The site-wise distribution of cases is shown in Table 1.

**Table 1:** Site-Wise Distribution

S.no	Site	Number of cases
1	Sinuses	58
2	Orbit	8
3	Lung	2
4	Bone	2
5	Brain	1
6	Small intestine	1
7	Nasopharynx	1
8	Scapula	1
9	Post cricoid	1
10	Hard palate	1
11	Pterygopalatine fossa	1

All the cases were analyzed by histopathological examination. The microscopy showed hyphae of mucor which were irregular, wide, aseptate, and with wide-angle branching. The hyphae were evident on H&E stains. However special stains such as PAS and GMS were done when needed. Most of the fungal hyphae were seen in areas of necrosis and some were seen within granulomas.

**Figure 1:**
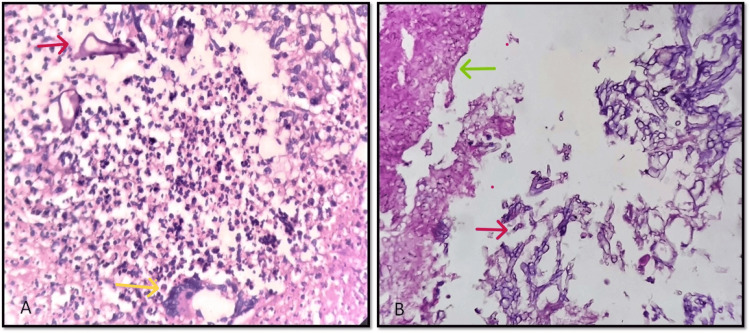
Granulomas and necrosis in mucormycosis A: Granulomas with giant cells (yellow arrow), mixed inflammatory cell collections, macrophages, and hyphae of mucormycosis. (Red arrow) (H&E, 400X) B: Areas of necrosis (green arrow) with hyphae of Mucormycosis (red arrow) which are irregularly wide and aseptate. (H&E, 100X)

Granulomatous inflammation was seen in 10 (14.5%) cases (Figure 1A). These granulomas were composed of macrophages, foreign body giant cells, lymphocytes, and a few neutrophils. Some of these granulomas showed central necrosis.

Fifty (72.4%) cases showed necrosis that was eosinophilic and with varying amounts of inflammation (Figure 1B). The percentage of necrosis varied from case to case, with some cases having large areas of necrosis and the percentage ranging from 5% to 90%.

Both acute inflammatory and chronic inflammatory cell collections were seen. Mixed inflammatory cell collections with the predominance of neutrophils were seen in 29 (42%) cases, chronic inflammatory cells with a predominance of lymphocytes were seen in 57 (82.6%) cases and eosinophils were seen in 14 (20.2%) cases.

**Figure 2:**
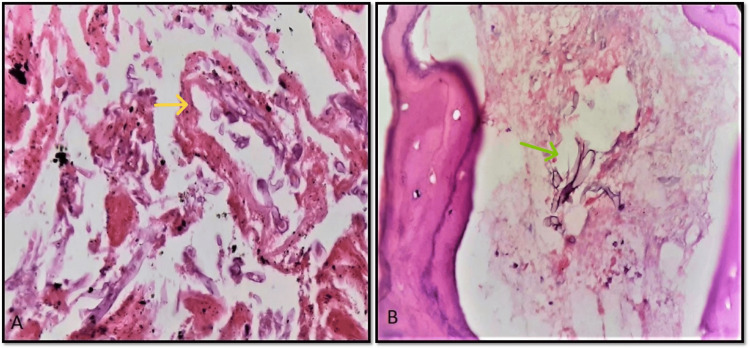
Angioinvasion and bone invasion by mucor hyphae A: Angioinvasion (yellow arrow) of mucor hyphae in necrotic areas, the hyphae are seen in the lumen and wall of blood vessels. (H&E, 400X) B: Bone invasion by mucor hyphae (green arrow) (H&E, 400X)

Angioinvasion was seen in 27 (39%) cases. The blood vessels were mostly seen in necrotic areas (Figure 2A) and in very few cases the blood vessels were seen in the viable tissue. The hyphae were seen in the lumen and walls of blood vessels. The bone invasion was seen in 26 (37.6%) cases. The hyphae were seen within the marrow (Figure 2B).

**Figure 3:**
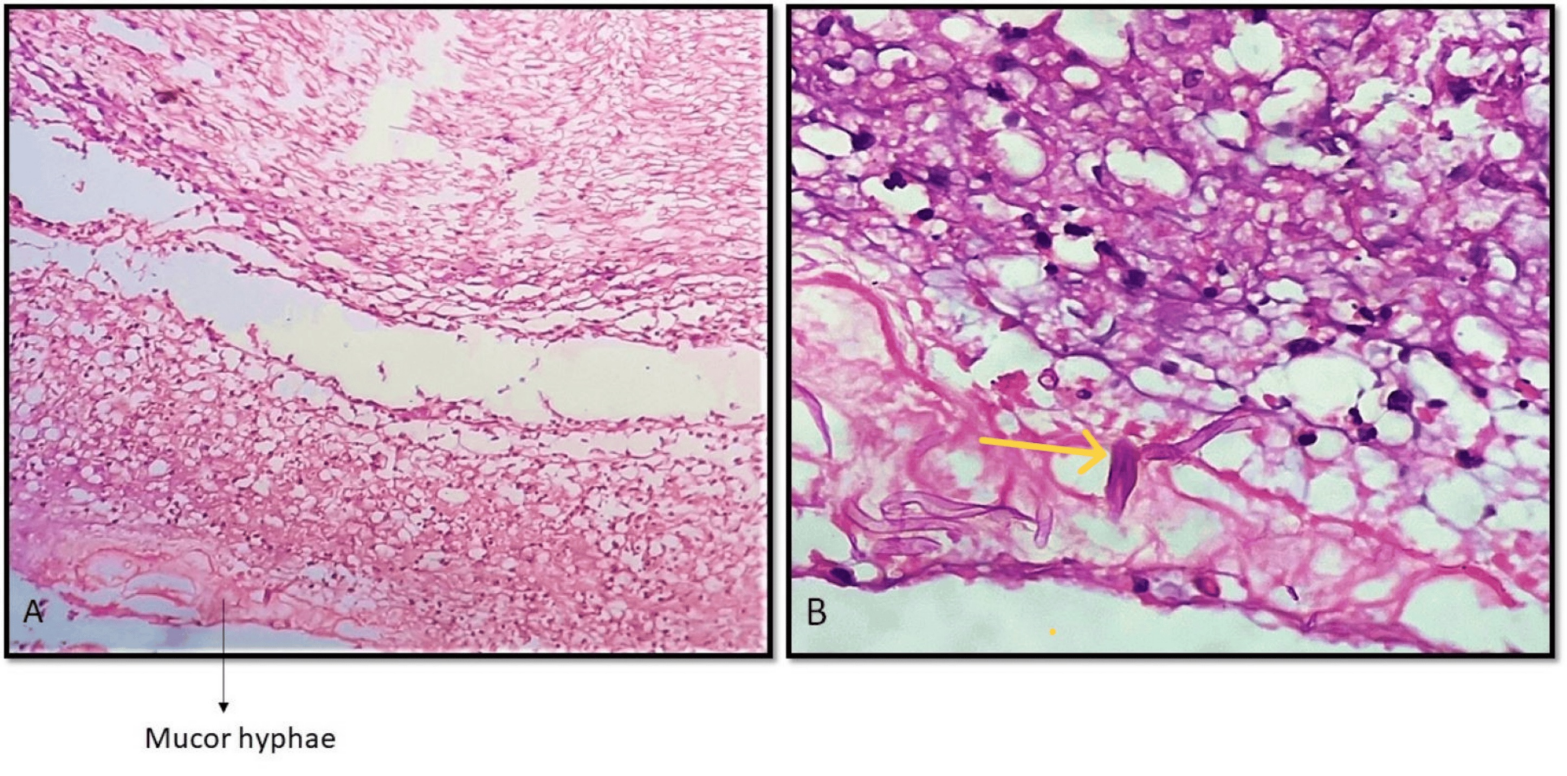
Perineural invasion by mucor hyphae A: Perineural invasion by mucor hyphae with necrosis and neutrophilic collections. (H&E, 100X) B: High power view of mucor hyphae (yellow arrow) with few neutrophils and necrosis (H&E, 400X)

Perineural invasion was seen in only one (1.4%) case involving the eyeball. Optic nerve invasion was also observed (Figures 3A, 3B).

There were three (4.3%) cases showing mixed fungal infection of mucor and aspergillus. All three were from the same maxillary sinus site.

**Figure 4:**
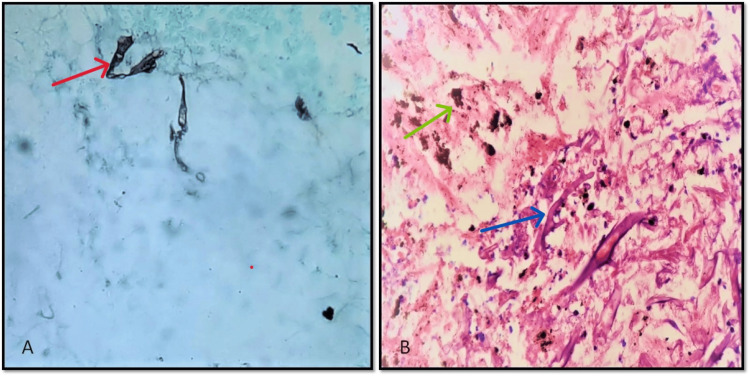
Mucor hyphae on GMS stain and lung tissue A: Mucor hyphae on Gomori methenamine silver stain (GMS) (Red arrow), these hyphae are grey-black in color (GMS, 400X) B: Mucor hyphae (blue arrow) in lung tissue, anthracotic pigment (green arrow) is seen with foci of necrosis and few neutrophils. (H&E, 400X)

Special stains such as GMS were done in two (2.8%) cases (Figure 4A) and PAS was done in one (1.4%) case.

KOH mount was done in 53 cases (53/69) of which 41 (77%) cases showed positivity and the remaining 12 (23%) cases were negative. Fungal culture was done in 56 cases (56/69) of which 38 (68%) cases were positive and 18 (32%) cases were negative.

## Discussion

In our study male preponderance was seen, constituting 50 (72%), the male-to-female ratio was 2.6:1 which was similar to studies done by Bala et al. [9], Roden et al. [7], Chavan et al. [10] and Sen et al. [11] showing 72%, 65%, 74.7%, and 100% respectively.

The age range in our study was 28 to 80 years and the mean age was 43 years which was similar to studies done by Chavan et al. [10] and Goel et al. [12]. In a study done by Bala et al. [9] mean age was 40.43 years and in studies done by Roden et al. [7] and Sen et al. [11], the mean age was 38.8 and 60.5 years respectively.

The most common underlying condition leading to mucormycosis in this study was diabetes mellitus, with 53 (77%) cases, many of whom had uncontrolled sugars. This was similar to studies done by Chavan et al. [10] and Goel et al. [12] where the incidence of diabetes was 86.6% and 94% respectively.

Paranasal sinuses (58, 84%) were the most common site of mucormycosis in our study with the majority being in the maxillary sinus. This was in contrast to studies done by Bala et al. [9] and Pagano et al. [13] where the common sites were rhino-orbital and pulmonary constituting 61.5% and 64% respectively.

Granulomas were seen in 10 (14.4%) cases. These are composed of lymphocytes, foreign body giant cells, and macrophages. In a study done by Goel et al. [12], granulomatous inflammation was seen in 11 (33%) cases which is comparable to our study. In a study done by Frater et al. [14] granulomas were seen in only 5% of cases. Necrosis was seen in 50 (72.4%) cases with varying percentages whereas in a study done by Goel et al. [12] necrosis was seen in 100% of cases with a percentage of necrosis ranging from 2 to 95%.

In our study, chronic inflammation comprising lymphocytes and plasma cells was seen in 57 (82.6%) cases and acute inflammation comprising neutrophils in 29 (42%) cases, whereas in a study done by Frater et al. [14] the predominant inflammatory response was neutrophils seen in 50% of cases.

Angioinvasion and bone invasion were seen in 27 (39%) and 26 (37.6%) cases whereas in a study done by Goel et al. [12] the percentages were 51% and 21% respectively. In a study done by Frater et al. [14], angioinvasion was seen in 100% of cases.

In the present study, perineural invasion was seen in only one (1.4%) case whereas in studies done by Goel et al. [12] and Frater et al. [14] perineural invasion was seen in 27% and 90% of cases which was very high compared to our study.

Rhino orbital mucormycosis was seen in eight (11%) of our cases with equal sex distribution, male-to-female ratio being 1:1, and a mean age was 47.8 years. In six out of eight cases post-COVID-19, one case was COVID-19 negative, and one case was COVID-19 positive. Four out of eight cases were diabetic whereas in a study done by Jiang et al. [15], rhino orbital cases showed a male preponderance and the mean age was 53.7 years with diabetes being the common risk factor. Perineural invasion of the optic nerve was seen in one of our rhino orbital cases, angioinvasion in eight cases, granulomas were seen in one case, and necrosis was seen in three cases. Angioinvasion in rhino orbital cases was also seen in a study done by Jiang et al. [15].

Two (2.8%) cases of mucormycosis were seen in the lung (Figure 4B). Each case involved one male and one female, with ages of 78 and 48 years, respectively. A similar study by Challa et al. [16] reported a total of seven patients, with a mean age of 46 years, showing a higher number of males. Both patients in our case also had diabetes mellitus, a condition also noted as the most frequent risk factor in Challa et al.'s [16] study. The lung cases presented with a neutrophilic infiltrate, necrotic areas, and fungal hyphae invading the blood vessels, characteristics also observed in Challa et al.'s research [16].

Mucormycosis was seen to be the most common fungus causing osteomyelitis and is more in the maxilla due to its proximity to paranasal sinuses [17]. We had two (2.8%) cases of fungal osteomyelitis, one involving calcaneum and the other being zygomatic bone, and both were post-COVID and diabetic.

We documented a case of cerebral mucormycosis, involving a woman with diabetes who was 44 years old. She came with a headache on left side and sinusitis that had lasted for 20 days. Medical imaging showed a clearly defined, mixed solid cystic growth in the left frontal lobe, which was also connected to the nasal cavity. The orbits on both sides were normal. In research conducted by Bala et al. [18], nine patients were identified with rhinocerebral mucormycosis, all of whom had diabetes, with an average age of 58.2 years.

Mucormycosis affecting the gastrointestinal tract is often found in the stomach before moving to the intestines. However, in our research, we encountered a 73-year-old male with mucormycosis in the small intestine, who initially showed symptoms of constipation and blockage in the intestines. A case report by Wotiye et al. [19] described mucormycosis in the colon of a 40-year-old male, who reported severe abdominal pain and a high fever. Another study by Thomson et al. [20] found that the stomach was a frequent location for mucormycosis, with symptoms including peptic ulcers.

We also noted mucormycosis in other sites such as the scapula, nasopharynx, hard palate, and pterygopalatine fossa.

Our study was limited by site-wise distrubution of mucor mycosis and our aim was to report incidence of cases. Follow-up of cases was not done.

## Conclusions

Mucormycosis manifested across various clinical sites, with a notable prevalence in paranasal sinuses. Additionally, it appeared in sites such as orbit, bone, lung, small intestine, brain, nasopharynx, scapula, post cricoid, hard palate, and pterygopalatine fossa. Necrosis was predominant among the cases, while granulomas were observed in the minority. Moreover, several cases exhibited angioinvasion, bone invasion, and optic nerve invasion consistent with findings from previous studies.
